# Contrasting effects of hyperoxia on GM-CSF gene transcription in alveolar epithelial cells and T cells

**DOI:** 10.14814/phy2.12324

**Published:** 2015-03-06

**Authors:** Anne Sturrock, Jessica A Baker, Mustafa Mir-Kasimov, Robert Paine

**Affiliations:** 1 Department of Veterans Affairs Medical CenterSalt Lake City, Utah, USA; 2 Division of Respiratory, Critical Care and Occupational Pulmonary Medicine, University of Utah School of MedicineSalt Lake City, Utah, USA

**Keywords:** Gene regulation, growth factors, hyperoxia, lung, transcription

## Abstract

Granulocyte/macrophage colony-stimulating factor (GM-CSF) is critically important for normal pulmonary innate immunity and for functional maturation of alveolar macrophages. Alveolar epithelial cells (AEC) are a major source of GM-CSF in the lung and express this growth factor constitutively, whereas most other cells, including T cells, express GM-CSF following inflammatory stimulation. AEC expression of GM-CSF is suppressed by oxidative stress, at least in part through induction of microRNA leading to increased mRNA turnover. In this report, we compare and contrast the effect of hyperoxia on transcriptional aspects of gene regulation of GM-CSF in lung epithelia and T cells of human and mouse origin. Similar to primary murine AEC, human H820 cells that express multiple characteristics of normal alveolar epithelial cells express GM-CSF constitutively, with decreased expression and increased mRNA turnover following exposure to hyperoxia. In contrast, hyperoxia induces augmented GM-CSF expression in human and murine activated T cells, in association with enhanced GM-CSF mRNA stability. Alveolar epithelial cells demonstrate constitutive transcription, with the proximal promoter in an open configuration in normoxia, without change in hyperoxia. Conversely, in both human and murine T cells, hyperoxia increased GM-CSF gene transcription. The proximal promoter was in a closed configuration in unstimulated T cells but became accessible upon activation and still more accessible in activated T cells exposed to hyperoxia. These fundamental differences in molecular regulation of GM-CSF expression highlight the distinctive niche of alveolar epithelial cell expression of GM-CSF and offer insights into the biology of GM-CSF in the setting of acute lung injury.

## Introduction

Alveolar epithelial cells (AEC) line the gas exchange surfaces of the lung and form an enormous interface with the external environment. They have important roles in gas exchange, as sources of basement membrane molecules in the lung, and in the production and recycling of pulmonary surfactant to lower surface tension in the lung. AEC also play critical roles in pulmonary innate immunity; in particular, AEC express molecules that help determine the mobility, accumulation, activation, and functional maturation of traditional inflammatory cells, such as alveolar macrophages, neutrophils, and dendritic cells. Granulocyte-macrophage colony-stimulating factor (GM-CSF, also known as colony-stimulating factor 2 or csf-2) is an endogenous pulmonary growth factor that is produced by AEC and activated T cells. GM-CSF has important paracrine effects on immune and inflammatory cells, especially alveolar macrophages (Huffman Reed et al. 1997; Paine et al. 2000, 2001, 2003; Shibata et al. 2001). In the absence of GM-CSF in the lung, normal functional maturation of alveolar macrophages is greatly impaired, resulting in a variety of host defense defects and in pathologic accumulation of surfactant proteins and lipids, severely impacting gas exchange.

A very large number of different kinds of insults can result in acute lung injury and its most severe form, the acute respiratory distress syndrome. Many of the conditions that lead to acute lung injury/acute respiratory distress syndrome involve oxidative stress. We have shown previously that murine AEC expression of GM-CSF is subject to suppression by exposure to oxidative stress in the form of hyperoxia, with important in vivo consequences, including increased susceptibility to lethal pneumonia and increased AEC apoptosis (Baleeiro et al. 2006). Decreased expression of GM-CSF by murine AEC during oxidative stress in vitro is at least in part a consequence of accelerated turnover of GM-CSF mRNA (Sturrock et al. 2010) as a result of the action of a specific microRNA family, miR133, directly affecting its stability (Sturrock et al. 2014). It remains unknown whether GM-CSF expression in AEC is also regulated at the level of transcription. T cells are also well-recognized sources of GM-CSF and can be induced to express high levels of GM-CSF with appropriate stimulation (Miyatake et al. 1988; Jenkins et al. 1995; Shannon et al. 1997). Under certain circumstances, expression of GM-CSF in these cells is also controlled at the level of mRNA stability (Bickel et al. 1990). However, it remains unknown if hyperoxia exerts any moderating effect on GM-CSF expression in T cells. This study was undertaken to investigate the hypothesis that the regulation of GM-CSF expression in the context of hyperoxia as a model of oxidative stress differs in important ways between T cells and AEC in both mice and humans. Detailed understanding of the regulation of GM-CSF expression in AEC and how it compares and contrasts with T cells increases the therapeutic opportunity for regulation of endogenous expression of this growth factor in the lung.

## Experimental Procedures

### Animals

Wild-type C57Bl/6 (Ly5.1; CD45.2) mice were obtained from Jackson Laboratory (Bar Harbor, ME). Mice were housed under specific pathogen-free conditions and were monitored daily by veterinary staff. The animal care committee at the Salt Lake City VA Medical Center approved all experiments.

### Isolation and purification of primary alveolar epithelial cells (AEC)

Primary murine type II AEC were isolated and purified using a modification of published methods (Corti et al. 1996; Mendez et al. 2006; Sturrock et al. 2014). Briefly, lung cells were released by intratracheal instillation of neutral protease (Dispase; Worthington Biochemical Corp, Lakewood, NJ). The cell suspension was subjected to sequential filtration and leukocytes were removed by positive selection with anti-CD32 and anti-CD 45 (BD Bioscience, San Jose, CA). Mesenchymal cells were removed from the cell suspension by overnight adherence to tissue culture-treated plastic (=day 0). Primary AEC were allowed to attach on dishes coated with fibronectin for 48 h, after which nonadherent cells were removed by gentle washing. Purity of the epithelial cell preparations was confirmed by staining with murine antivimentin to identify mesenchymal and bone-marrow-derived cells. Routinely, fibroblast contamination was 3–6% on day 3 after isolation.

### Cell lines

EL4 cells (a murine T lymphocyte line), Jurkat cells (a human T lymphocyte line), and NCI-H820 cells [H820] (ATCC® HTB181™) (a human lung adenocarcinoma cell line with-type II cell characteristics), were obtained from ATCC (Manassas, VA). The cell lines were maintained in RPMI 10% FCS (EL4 and Jurkat) or RPMI 5% FCS (H820). The original cultures were expanded and stocks frozen within 7 days of receipt to maintain a supply of close to original cells. Cultures were replenished with a new stock every 4–6 weeks.

### In vitro exposure to hyperoxia

#### Human H820 cells and primary murine AEC

In order to model the stress of sublethal in vivo hyperoxia in an in vitro system, human H820 cells and primary murine AEC were exposed to an atmosphere of 80% oxygen/5% CO_2_ for 48 h. Cells were placed in a sealed self-contained chamber (Billups- Rothenberg, Del Mar, CA). This chamber was humidified, maintained at 37°C and flushed daily with a commercially available gas mixture of CO_2_ and oxygen, adjusted to maintain a fractional concentration of oxygen of 0.80 as measured in real time with an oxygen analyzer within the chamber (maxO2+, Maxtec, Salt Lake City, UT).

#### EL4 and Jurkat T cells

Both T-cell lines were exposed to hyperoxia for 48 h as described above. During the last 16 h of hyperoxia exposure the T cells were activated by the addition of phorbol myristate acetate (PMA) (20 ng/mL) and A23187 (1 *μ*mol/L) as described by Cockerill et al. (1999).

### Real-time (RT)-PCR

Total cellular RNA was isolated, first strand cDNA was reverse transcribed and specific amplification in a PikoReal Thermocycler (ThermoFisher, Waltham, MA) was performed using a 2-step cycle program (Tm = 60°C) with dissociation analysis. Gene-specific primers were designed using the Roche Applied Science Universal Probe Library Assay Design Center. A “no template control” was included in each experiment. Each biological sample was amplified in duplicate and the average of the duplicates taken for statistical analysis. The threshold cycle from GAPDH was used as a calibrator to normalize the specific RNA. Results are expressed as fold-change over control values after correcting for GAPDH and setting the first biological control at 100%.

### GM-CSF protein measurement

Cells were cultured in the appropriate growth media with and without stimuli as detailed above or in the results. After stimulation (e.g., 32 h in hyperoxia) fresh media were added to the cells and stimulation continued for a further 16 h. This media were collected, immediately centrifuged to remove cell debris and either immediately assayed for cytokines levels by ELISA (R&D Systems, Minneapolis, MN), or frozen in suitable aliquots at −80°C and for later assay with at most a single freeze–thaw.

### Determination of GM-CSF mRNA stability

Studies of comparative mRNA stability were carried out in cells exposed to normoxia or hyperoxia (48 h) as previously described (Sturrock et al. 2010). In human H820 and primary murine AEC cells, mRNA decay was estimated in cells in control medium. Because Jurkat and EL4 T cells produce little GM-CSF mRNA at baseline, these cells were activated with PMA/A23187 (see above) for the last 16 h of hyperoxia. To assess mRNA turnover, actinomycin D (5 *μ*g/mL) was added to stop transcription, and relative mRNA expression (RT-PCR) was determined over time. Time 0 was collected immediately before addition of actinomycin D. Precise time points were achieved by rapid solubilization in RNA lysis buffer. Data are expressed relative to time 0 for each experimental condition.

### Relative GM-CSF transcription in normoxia and hyperoxia

Following transcriptional activation, unspliced nuclear RNA (nRNA) accumulates transiently in the nucleus providing a surrogate of transcription rate. We estimated the rate of transcription of the GM-CSF gene by a method using real-time polymerase chain reaction and an intron-specific probe (Lipson and Baserga 1989; Chivers et al. 2006; Newton et al. 2010). The TaqMan™ (Life Technologies, Grand Island, NY) probe and primer set was designed to cross the 5′ splice junction between each intron and the preceding exon in GM-CSF, exon 1/intron A. The level of unspliced GM-CSF nRNA was normalized to U6 small nRNA (RNU6). Similar results for probe and primer sets crossing every 5′ exon/intron junction of the GM-CSF gene have been described (Newton et al. 2010). RNA samples were subject to RT-PCR in both the presence and the absence of the reverse transcriptase in order to estimate contamination by genomic DNA. The levels of contaminating genomic DNA were found to be consistent and low (<10%) in all samples (data not shown). Any sample with >10% genomic contamination for RNU6 was excluded.

RNA extraction, cDNA synthesis, and RT-PCR were performed as described above and using the following primers:

Mouse GM-CSF (csf2)

Forward: AGAAGCCCTGAACCTCCTGGATG
Reverse: CCTGGGAACAACCCAACTCTCT
Probe: 5-6FAMCCTGTCACGTTGGTGAGTGA- MGB-3

Human GM-CSF (CSF2)

Forward: GCGTCTCCTGAACCTGAGTAGAG
Reverse: GGCACAGGCCCACATTCT-5
Probe: 5-6FAMTGCTGCTGAGATGGTAAGTGA-MGB-3

Mouse/Human RNU6

Forward: AATTGGAACGATACAGAGAAGATTAGC
Reverse: GGAACGCTTCACGAATTTGC
Probe: 5-6FAM-TGGCCCCTGCGCAA-MGB

### Accessibility of the GM-CSF proximal promoter

Analysis was performed using an EpiQ kit (Bio-Rad, Hercules, CA) as per the manufacturer's recommendation. Primary murine AEC cells and human H820 cells were seeded into 48-well plates and allowed to attach for 48 h. The cells were then washed three times with PBS and maintained either in normoxia or hyperoxia. Both T-cell lines were maintained at a concentration of 10^6^/mL and were exposed to normoxia or hyperoxia for 48 h prior to assay. In order to activate T cells for GM-CSF expression, cultures were stimulated with PMA/A23187 or left in media alone for the final 16 h of the experiment, as described above. After these incubations, cells were permeabilized with a weak detergent and incubated with or without DNase for 1 h at 37°C. Genomic DNA was isolated with a DNeasy kit (Qiagen, Valencia, CA) and proximal promoters for human and mouse GM-CSF and GAPDH amplified by RT-PCR using the following program:

Step 1: 1 min at 96°C
Step 2: 15 sec at 96°C
Step 3: 1 min at 67°C
Step 4: 30 sec at 80°C
Steps 2–4 were repeated for 40 cycles.

RT-PCR of human GM-CSF and GAPDH proximal promoters was performed at 72°C for Step 3. A dissociation curve was performed for every analysis to ensure a single melt curve for each primer pair.

The accessibility of each gene was determined by the ratio of Ct digested/Ct undigested, with a higher ratio indicating increased accessibility of the DNA in question. Primers were designed to prime within a DNase hypersensitive site located inside the proximal promoter. Primers to analyze the proximal promoters of human and mouse GM-CSF genes were designed as recommended by the manufacturer. The UCSC Genome Bioinformatics site was used to identify the upstream promoter sequence of the GM-CSF gene. Primer3, a primer design tool from MIT, was used to design and select appropriate primer sets for use in RT-PCR analysis with the EpiQ kit. The specificity of the primer pair was verified using in silico on the UCSC Genome Bioinformatics site. Finally, an optimal melt temperature for each primer set was determined using dissociation curve analysis.

The following primer sets were used:

Mouse GM-CSF (csf2)

Forward: CAGAAGGTGGCTGGAAAGAGAACGGG
Reverse: GCTGGGGCGGGGTTTGGGACATACT

Human GM-CSF (CSF2)

Forward: CGGGTGGGTGGGCTGTCGGTTCTTG
Reverse: GGCCACAGTGCCCAAGAGCAGCAGG

Primers for analysis of mouse and human GAPDH promoters were supplied in the Bio-Rad EpiQ kit. GAPDH is used as an example of an open gene.

### Statistical analysis

Data are presented as mean ± standard error of the mean (SEM). Statistical analysis was carried out using GraphPad Prism v4C software (GraphPad, Inc., La Jolla, CA). Differences between two groups were compared using the unpaired Student's *t*-test. Two-tailed tests of significance were used throughout. Differences between multiple groups were compared with one-way analysis of variance. Comparisons were deemed statistically significant for *P* < 0.05.

## Results

### Effect of hyperoxia on GM-CSF expression by human and murine lung epithelial cells

**Figure 1:**
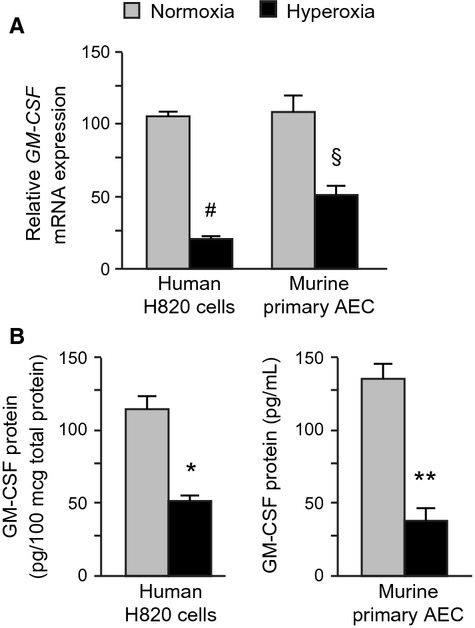
Effect of exposure to hyperoxia on lung epithelial cell expression of GM-CSF. Human H820 cells and primary murine AEC were exposed to hyperoxia (80% oxygen/5% CO_2_; black bars) or normoxia (21% oxygen/5% CO_2_, gray bars) for a total of 48 h. After 32 h, the culture medium was replaced with fresh medium, and the cells continued in hyperoxic or normoxic conditions for 16 h further for determination of GM-CSF mRNA in cell lysates and GM-CSF protein in the culture supernatant. GM-CSF mRNA was measured by RT-PCR, normalized to GAPDH levels and expressed relative to the first sample in normoxia (100%) (A). GM-CSF protein in the culture supernatants was measured by ELISA (R&D Systems). Data are expressed as pg GM-CSF/ml (B). All data are expressed as means ± SEM, *n* = 3, and are representative of three independent experiments. ^#^*P* < 0.0001; ^§^*P* = 0.011; *P < 0.001; **P < 0.01.

Human H820 cells express multiple characteristics of human alveolar epithelial cells and have been used as a model of type II alveolar epithelial cells in vivo (O'Reilly et al. 1989). These cells express abundant GM-CSF at baseline, with significant suppression of both mRNA and protein expression during culture in hyperoxic conditions (Fig.1). This pattern strongly resembles that which we have reported previously in primary murine AEC, with constitutive expression in normoxic conditions and significant suppression by hyperoxia (Sturrock et al. 2010). In both cells, the effect of hyperoxia on mRNA expression was mirrored by changes in secreted protein. These data demonstrate strong similarities between human and murine alveolar epithelial cells with respect to GM-CSF expression and confirmed that the observations concerning the effect of hyperoxia on this expression could be extended to human cells.

### Effect of hyperoxia on GM-CSF expression by human- and murine-activated T cells

**Figure 2:**
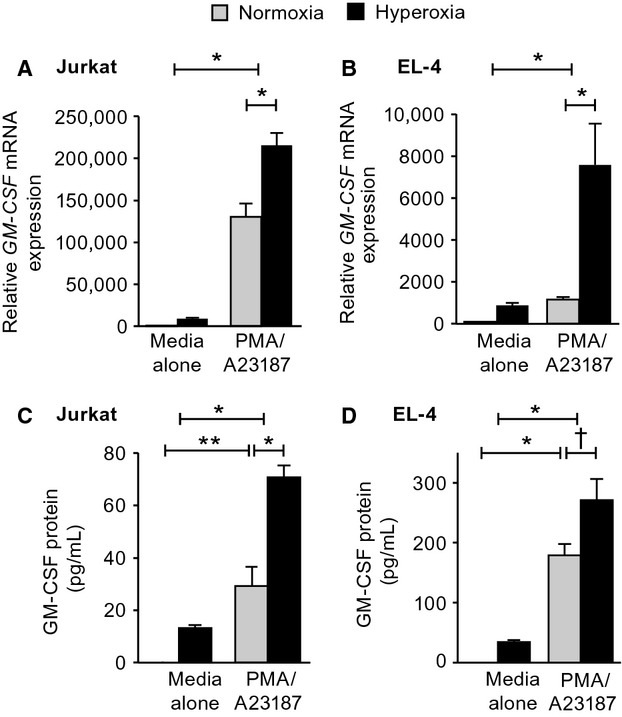
Effect of exposure to hyperoxia on expression of GM-CSF by T-cell lines. Human (Jurkat) and mouse (EL4) T cells were exposed to hyperoxia (80% oxygen/5% CO_2_; black bars) or normoxia (21% oxygen/5% CO_2_; gray bars) for a total of 48 h. After 32 h, the culture medium was then replaced with media alone or media containing PMA/A23187 as described in Methods. The cells were continued in hyperoxic or normoxic conditions for 16 h further. GM-CSF mRNA was determined in cell lysates and GM-CSF protein measured in the culture supernatants. GM-CSF mRNA was assessed by RT-PCR, normalized to GAPDH levels and expressed relative to the first sample of normoxia (100%) (A and B). GM-CSF protein in the culture supernatants was measured by ELISA. Data are expressed as pg GM-CSF protein/ml (C and D). All data are expressed as means ± SEM, *n* = 3, and are representative of three independent experiments. *P < 0.001; **P < 0.01.

We next determined whether hyperoxia exerted the same suppressive impact on GM-CSF expression in human and murine T cells. There is a considerable body of work elucidating the genetic components required for GM-CSF gene expression upon activation of T cells (reviewed in Shannon et al. 1997). As anticipated (Bickel et al. 1990; Cockerill et al. 1999), unactivated human Jurkat and mouse EL4 T cells express very low levels of GM-CSF mRNA (Fig.2A and B) and no GM-CSF protein (Fig.2C and D). In response to activation with PMA and A23187, both cells were rapidly induced to produce GM-CSF mRNA and protein (Fig.2A–D, Jenkins et al. 1995; Osborne et al. 1995). Exposure of human and murine T cells to hyperoxia alone, in the absence of other stimulation, resulted in modest induction of GM-CSF expression. However, if T cells from either species were cultured in hyperoxia prior to activation with PMA/A23187, GM-CSF mRNA expression was significantly increased compared to cells activated in normoxia. These changes were reflected in increased GM-CSF protein expression when cells were activated in hyperoxia compared to similarly activated cells cultured in normoxia. The increase in GM-CSF express in response to hyperoxia was similar in both human and murine T cells and was in stark contrast to the pattern in murine AEC and human H820 cells.

### Effect of hyperoxia on the stability of GM-CSF mRNA in lung epithelia compared with activated T cells

**Figure 3:**
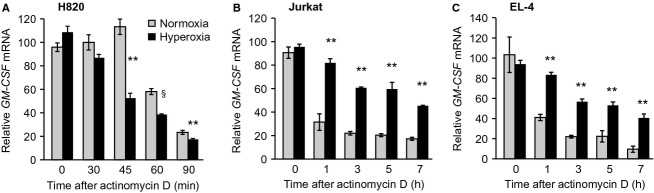
Effect of exposure to hyperoxia on GM-CSF mRNA stability. Human H820 cells (A) and PMA/A23187 activated Jurkat (B) and EL4 (C) T cells were placed in normoxia (gray bars) or hyperoxia (black bars) for 48 h, after which actinomycin D (5 *μ*g/mL) was added to stop transcription (time 0). Relative mRNA for GM-CSF was determined at time points from 0 to 90 min (H820) or from 0 to 7 h (Jurkat and EL4) after addition of actinomycin D. mRNA levels were measured by RT-PCR and normalized to GAPDH, with zero time in normoxia considered 100%. Data are expressed as mean ± SEM, *n* = 3. Each experiment is representative of two independent experiments. **P < 0.01; ^§^*P* = 0.033

Having determined previously that mRNA turnover plays an important role in the regulation of GM-CSF expression in murine primary AEC during hyperoxic stress, we next determined the role of mRNA stability in GM-CSF regulation in human H820 cells and in murine and human T cells. Similar to primary murine AEC (Sturrock et al. 2010), exposure of H820 cells to hyperoxia resulted in more rapid turnover of GM-CSF mRNA (Fig.3A). Forty-five minutes after translation was blocked by the addition of actinomycin D, relative GM-CSF mRNA levels were greatly reduced in cells subjected to 80% oxygen for 48 h compared with cells in room air. This difference persisted through 90 min, indicating that hyperoxia accelerated the turnover of GM-CSF mRNA in this human lung epithelial cell line. In initial experiments, we found that GM-CSF mRNA is more stable in activated T cells compared to alveolar epithelial cells in both species. Therefore, we used a longer time course following actinomycin treatment to assess mRNA turnover in T cells. In marked contrast to the situation in H820 cells, exposure to hyperoxia resulted in stabilization of GM-CSF mRNA in both Jurkat (Fig.3B) and EL4 T cells (Fig.3C). This result demonstrates a fundamental difference in the regulation of GM-CSF expression in response to stress between alveolar epithelial cells and T cells.

### Effect of hyperoxia on the rate of GM-CSF transcription

**Figure 4:**
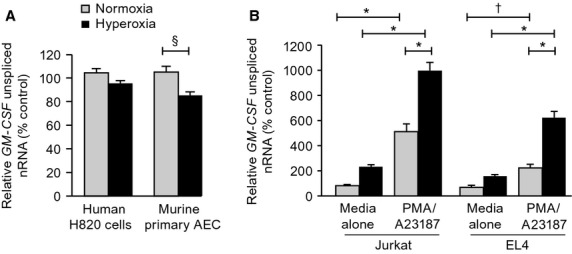
Effect of exposure to hyperoxia on GM-CSF transcription rate. Lung epithelial cells (A) and T cells (B) were exposed to normoxia (gray bars) or hyperoxia (black bars) for 48 h in control medium. The relative rate of transcription of GM-CSF was determined by real-time PCR using primers designed to span the junction of Exon 1/Intron A of human and murine GM-CSF genes (see Methods). Values are normalized to rate of expression of RNu6 nRNA. Data are expressed as mean ± SEM, *n* = 3. The experiment shown is representative of two independent experiments. ^§^*P* = 0.027; *P <0.001; ^†^*P* < 0.05.

Having determined the different contributions of changes in mRNA stability to the regulation of GM-CSF expression in alveolar epithelial cells compared to T cells, we next determined whether additional transcriptional effects were also involved in either group of cells. We first measured unspliced exon/intron junction nRNA corresponding to GM-CSF exon 1/intron A (ex1/IA) in unstimulated H820 and primary AEC under basal conditions in normoxia and hyperoxia. Hyperoxia did not affect the RT-PCR amplification levels of GM-CSF ex1/IA normalized to RNu6 in either epithelial cell compared to cells in normoxia, indicating that the rate of GM-CSF gene transcription was not altered by hyperoxia in either instance (Fig.4A). We next assessed the rate of transcription of the GM-CSF gene in our model T cells. In contrast with human and murine alveolar epithelial cells, in unstimulated human Jurkat and murine EL4 T cells cultured in normoxia, amplification of nRNA using primers amplifying ex1/IA resulted in relative levels that were minimally different from those obtained in the absence reverse transcriptase, suggesting that the gene was not actively transcribed in unstimulated cells. Activation of both Jurkat and EL4 cells with PMA/A23187 resulted in a significant increase in relative levels of GM-CSF ex1/IA (Fig.4B), indicating induction of gene transcription. Exposure of Jurkat and EL4 cells to hyperoxia followed by activation with PMA/A23187 resulted in synergistically increased levels of GM-CSF ex1/IA normalized to RNu6 (Fig.4B) compared to cells stimulated in normoxia. Together these data suggest that regulation of GM-CSF expression in murine and human T cells is transcriptionally regulated and that exposure to hyperoxia significantly augments this transcription, further emphasizing the differences in the mechanisms regulating GM-CSF expression in alveolar epithelial cells and T cells.

### Accessibility of the GM-CSF proximal promoter in normoxia and hyperoxia

**Figure 5:**
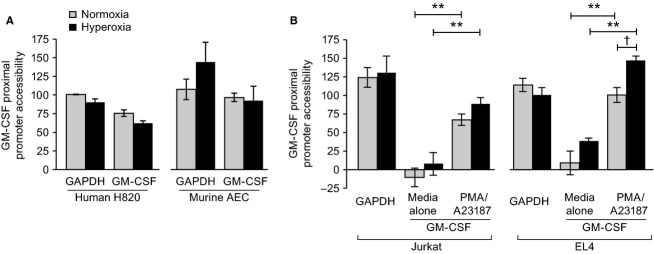
Accessibility of GM-CSF and GAPDH proximal promoters. Cells were exposed to normoxia or hyperoxia (80% oxygen) for 48 h in media alone. Chromatin DNA was isolated as outlined in *Methods* from cells, treated or untreated with DNase. RT-PCR of human and murine GM-CSF and GAPDH proximal promoters were performed as described to provide an estimate of proximal promoter accessibility. Lung epithelial cells (human H820 cells and primary murine AEC, A) were cultured in media alone. T-cell lines (human Jurkat cells and murine EL4 T cells, B) were cultured in media alone or were exposed to PMA (20 ng/mL) and A23187 (1 *μ*mol/L) for the last 16 h of the experiment. Data are analyzed as the Ct differential between the DNase digested sample and undigested sample. The value for GAPDH, an accessible housekeeping gene, under normoxic (and in T cells unactivated) growth conditions was assigned a value of 100%. Data are the mean ± SEM of three individual experiments. **P < 0.001; ^†^*P* < 0.05

The observations that lung epithelial cells transcribe GM-CSF at baseline and that the rate of transcription is unaffected by hyperoxia, whereas T cells do not transcribe GM-CSF unless activated and the rate transcription is enhanced with hyperoxia may be a reflection of differences in the accessibility of the GM-CSF proximal promoter in the two cell types. To evaluate promoter accessibility, we measured the vulnerability to DNase (MNase) digestion of the proximal promoter region of the murine GM-CSF gene in the four cell types (Holloway et al. 2003). In unstimulated human H820 and murine AEC the proximal promoter of GM-CSF showed a degree of accessibility to nuclease digestion similar to that of GAPDH, an epigenetically “open” gene (Fig.5A). The accessibility to nuclease digestion of the GM-CSF proximal promoter region did not change in response to exposure to hyperoxia in either epithelial cell. In contrast, similar assessment of the accessibility of the GM-CSF proximal promoter in Jurkat and EL4 T cells demonstrated that activation of these cells was required for accessibility (Fig.5B). Moreover, exposure of T cells to hyperoxia without additional activation resulted in an increased degree of accessibility of the GM-CSF promoter (Fig.5B) consistent with our data above showing that these conditions were sufficient to induce GM-CSF expression in these cells (see Fig.2). Finally, the combination of hyperoxia and activation with PMA/A23187 resulted in still further increase in promoter region accessibility, especially in EL4 cells (Fig.5B). Thus, in both lung epithelial and T cells there is a concordance between GM-CSF expression and accessibility of the proximal promoter. In alveolar epithelial cells the promoter is accessible and there is active transcription of the GM-CSF gene under basal conditions in normoxia, without change in hyperoxia. In our T-cell models, the promoter is only accessible after activation, leading to active transcription. In those cells, exposure to hyperoxia prior to activation results in an accessible promoter confirmation and ongoing transcription.

## Discussion

GM-CSF plays a crucial role in homeostasis and host defense in the lung. Although a number of different cell types can express GM-CSF, alveolar epithelial cells are a major source of this growth factor in the lung, with regulation of expression that has important consequences for the integrity of the alveolar space and pulmonary host defense in the setting of acute lung injury. In previous studies, we determined that alveolar epithelial cell GM-CSF expression is greatly suppressed in the setting of oxidative stress, at least in part as a consequence of changes in GM-CSF mRNA stability (Baleeiro et al. 2006; Sturrock et al. 2010). In this study, we have expanded the understanding of the distinctive molecular regulation of GM-CSF in alveolar epithelial cells and have demonstrated a marked contrast between these cells and T cells. In alveolar epithelial cells, the proximal promoter region is in an open configuration, with ongoing transcription at baseline. In response to oxidative stress, alveolar epithelial cells demonstrate no change in GM-CSF transcription or promoter accessibility, despite suppression of mRNA and protein expression. In contrast, GM-CSF expression in T cells requires activation, which results in transition of the proximal promoter from a closed to an open configuration and induction of transcription. Rather than suppressing expression, hyperoxia results in further induction of expression and stabilization of GM-CSF mRNA. Importantly, we found that the molecular regulation of GM-CSF expression was similar in murine and human cells for both alveolar epithelial cells and T cells.

Studies involving GM-CSF mutant mice, receptor mutant mice, antibody neutralization of GM-CSF and targeted replacement of GM-CSF within the lung have elucidated the role of GM-CSF in pulmonary homeostasis (Dranoff et al. 1994; Nishinakamura et al. 1995; Huffman et al. 1996; Ikegami et al. 1996; Reed and Whitsett 1998; Zsengeller et al. 1998; Reed et al. 1999; Bozinovski et al. 2004). These studies demonstrate that alveolar macrophages have an absolute requirement for GM-CSF for induction and maintenance of functional maturation. Loss of GM-CSF expression, even for short periods, results in an immature phenotype with impairment of macrophage clearance of pulmonary surfactant, leading to accumulation of surfactant phospholipid and protein in the lungs and the pathologic picture of alveolar proteinosis (Bozinovski et al. 2002). In the absence of GM-CSF, mice are more susceptible to pneumonia from a variety of pathogens (LeVine et al. 1999; Paine et al. 2000; Shibata et al. 2001; Steinwede et al. 2011) and demonstrate impaired healing and more severe fibrosis after acute injury with bleomycin or intratracheal fluorescein isothiocyanate (FITC) (Christensen et al. 2000; Moore et al. 2001). In addition to its role as a paracrine factor in the lung, GM-CSF is an autocrine factor for AEC, acting as a mitogen and anti-apoptotic factor (Huffman Reed et al. 1997; Reed and Whitsett 1998; Paine et al. 2000, 2003). Enhanced expression of GM-CSF in the lung preserves alveolar epithelial cells and provides significant protection in models of acute lung injury or infection (Paine et al. 2000, 2003).

The alveolar epithelium is a major source of GM-CSF in the lung. Alveolar epithelial cells express abundant GM-CSF constitutively, with further induction in response to select inflammatory stimuli, such as IL-1 (Sturrock et al. 2010; Mir-Kasimov et al. 2012). In contrast, most other cells that express GM-CSF, including T cells, require stimulation to induce this expression (Shi et al. 2006). Alveolar epithelial cell expression of GM-CSF is greatly suppressed in the setting of oxidative stress in vivo or in vitro. Short term exposure of mice to sublethal hyperoxia results in diminished alveolar epithelial cell GM-CSF expression, leading to impaired alveolar macrophage function and increased susceptibility to pneumonia (Baleeiro et al. 2006). The interplay between oxidative stress and GM-CSF expression is particularly evident in the setting of chronic ethanol ingestion, a circumstance that induces chronic oxidative stress in the alveolar space and in which epithelial barrier dysfunction may be reversed by treatment with GM-CSF (Guidot and Roman 2002; Pelaez et al. 2004). These observations provide strong motivation for better understanding of the details concerning the cell-specific molecular regulation of GM-CSF in alveolar epithelial cells.

Several considerations support the value of investigating the details of GM-CSF regulation in alveolar epithelial cells and the differential response of alveolar epithelial cells and T cells to oxidative stress. GM-CSF in the alveolar space is essential for normal alveolar homeostasis and acts on alveolar epithelial cells to promote proliferation and inhibit apoptosis due to mitochondrial stress. Thus, understanding the details of GM-CSF regulation in alveolar epithelial cells during oxidative stress may offer important insights into pathophysiology and new opportunities for therapeutic intervention. T cells are a recognized source of GM-CSF in the setting of acute and chronic inflammation and are a cell for which regulation of GM-CSF expression in general has been investigated in some detail. The distinct regulation of GM-CSF in alveolar epithelial cells is best appreciated in contrast with that in T cells. The differential response of GM-CSF expression between these cell types indicates that changes in gene expression in response to hyperoxia are determined by the cellular context. The induction of T-cell GM-CSF expression in T cells during hyperoxia also offers the possibility that, under appropriate circumstances, immune cell GM-CSF could provide an element of protection in the lung when alveolar epithelial cell expression is diminished due to inflammatory lung injury.

In general, expression of GM-CSF involves control at both the transcriptional and posttranscriptional levels. The GM-CSF gene contains a well-characterized proximal promoter region as well as an upstream enhancer; together these contain the elements required for response to most activators of GM-CSF (Cockerill et al. 1993, 1995). In particular, T cells have been the focus of extensive study and the specific promoters regulating GM-CSF expression by T cells are well characterized (Shannon et al. 1997). Posttranscriptional control of GM-CSF is largely mediated at the level of mRNA stability. AU-rich sequences in the 3′ untranslated region of the mRNA have been implicated in the precise regulation of turnover of GM-CSF mRNA (Iwai et al. 1991). We recently reported that the miR133 microRNA family, which is induced in murine AEC in the setting of hyperoxia, suppresses GM-CSF expression through direct interaction with sequences in this 3′-untranslated region to decrease mRNA stability (Sturrock et al. 2014).

There is a stark contrast with respect to GM-CSF regulation between alveolar epithelial cells in both human and mouse compared to T cells in the same species. As might be anticipated based on constitutive expression in the epithelial cells, the proximal promoter of GM-CSF in these cells is in an open configuration with evidence of ongoing transcription. Conversely, in T cells, which express little or no GM-CSF at baseline, the proximal promoter is relatively closed until the cells are stimulated, when it shifts to an open configuration and transcription is induced. The response to hyperoxia also differs markedly between the cell types. Hyperoxia destabilizes GM-CSF mRNA in alveolar epithelial cells, resulting in significantly suppressed GM-CSF expression, with no change in GM-CSF transcription. In contrast, in T cells, hyperoxia enhances promoter openness and transcription, while stabilizing GM-CSF mRNA. These differences explain the opposite effects of oxidative stress on the two cell types and have important implications for the response of the lung to this common stress.

There are a several important considerations with respect to our experimental design. We have used primary murine AEC to model the behavior of alveolar epithelial cells in the lung. Exposure of mice to hyperoxia in vivo results in suppression of whole lung GM-CSF mRNA expression and suppression of GM-CSF mRNA expression in the alveolar wall, as determined by laser capture microdissection and RT-PCR (Baleeiro et al. 2006). This result is confirmed in freshly isolated type II AEC taken from the lungs of mice following in vivo exposure to hyperoxia (Sturrock et al. 2014). The dose response to hyperoxia differs between intact mice and cells in vitro. As in previous studies, we have used in vitro exposure to 80% oxygen to induce oxidative stress modeling the results from exposure of mice in vivo to >90% oxygen (Sturrock et al. 2010, 2012). H820 cells have been used as effective models of human type II alveolar epithelial cells, based on maintenance of classic type II cell characteristics (surfactant protein and lipid expression, lamellar body and microvilli) in cell culture (O'Reilly et al. 1989). The similarities between primary murine AEC and H820 cells with respect to the regulation of GM-CSF expression in hyperoxia strongly suggest that our results are not specific for mice but are applicable to humans as well. The parallel details in murine and human T cells provide further indication that our comparison reflects regulatory differences between cell types rather than differences between species.

To assess GM-CSF gene transcription, we used a RT-PCR method that examined the steady-state amount of GM-CSF mRNA precursor [unspliced nuclear RNA (nRNA)] (Lipson and Baserga 1989; Chivers et al. 2006). Previous studies with a lung cancer cell line (A549 cells) have demonstrated that measurement of unspliced GM-CSF nRNA may be used as a surrogate for transcription rate (Newton et al. 2001). The ability to amplify the signal with RT-PCR is a significant advantage that gives reliable results by increasing the signal to-noise ratio and allows for relative transcription assessment in primary cells were material is limiting.

In order to compare accessibility GM-CSF proximal promoter for transcription among the cell types, we used a recently developed method in which susceptibility of the proximal promoter of the GM-CSF gene to nuclease (MNase) digestion is used as a measure of openness. The degree of accessibility of the GM-CSF promoter was normalized to the accessibility of the “fully open” GAPDH promoter. This assay is based on the assumption that MNase susceptibility under nondenaturing conditions is reflective of a promoter configuration permissible for transcription element(s) binding. We analyzed the chromatin structure of the first 300 bp upstream of the start ATG of human and mouse GM-CSF genes using the UCSC Genome Bioinformatics site. The first 120 bp upstream of the ATG site contains three conserved functional domains of GM-CSF (in both human and mouse) that are required for GM-CSF maximum gene activation in T cells, fibroblast, and endothelial cells (Shannon et al. 1997). Moreover, deletion analysis of the mouse GM-CSF promoter identified maximum induction with PMA/A23187 within the first 226 bp upstream of ATG (Miyatake et al. 1988). Thus, we are confident that the proximal promoter of GM-CSF is within the region of analysis.

In summary, we have shown that the impact of oxidative stress on expression of GM-CSF differs widely between alveolar epithelial cells and T cells in both mouse and humans. Hyperoxia results in greatly reduced GM-CSF expression by alveolar epithelial cells as a consequence posttranscriptional effects, with little change in promoter accessibility or constitutive transcription. In contrast, T cells expression is regulated at both transcriptional and posttranscriptional levels, with promoter accessibility increased by both activation and hyperoxia. Furthermore, GM-CSF mRNA is stabilized in the setting of hyperoxic stress, further increasing expression. Understanding the cell-specific differential response to oxidative stress has important implications for stress responses in the lung in the setting of lung injury and for therapeutic manipulation of GM-CSF expression to enhance pulmonary host defense and preserve the integrity of the alveolar space.
